# Examining Health-Seeking Behavior among Diverse Ethnic Subgroups within Black Populations in the United States and Canada: A Cross-Sectional Study

**DOI:** 10.3390/ijerph21030368

**Published:** 2024-03-19

**Authors:** Yordanos M. Tiruneh, Oluwatunmininu Anwoju, Ariel C. Harrison, Martha T. Garcia, Shauna K. Elbers

**Affiliations:** 1 School of Medicine, University of Texas at Tyler, Tyler, TX 75708, USA; 2 Division of Infectious Diseases, Department of Internal Medicine, University of Texas Southwestern Medical Center, Dallas, TX 75390, USA; 3 HCA Houston Healthcare, Kingwood, TX 77339, USA; oluwatunmininu.anwoju@hcahealthcare.com; 4 School of Public Health, University of Texas Health Science Center at Houston, Houston, TX 77030, USA; ariel.c.harrison@uth.tmc.edu; 5 School of Medicine, University of Texas Medical Branch, Galveston, TX 77555, USA; mtgarcia@utmb.edu; 6 School of Interdisciplinary Arts and Sciences, University of Washington Bothell, Bothell, WA 98011, USA; ske9902@uw.edu

**Keywords:** African American, African, Caribbean, healthcare utilization, ethnic disparities, health-seeking behavior, intra-group difference, black populations, ethnic subgroups

## Abstract

The Black populations, often treated as ethnically homogenous, face a constant challenge in accessing and utilizing healthcare services. This study examines the intra-group differences in health-seeking behavior among diverse ethnic subgroups within Black communities. A cross-sectional analysis included 239 adults ≥18 years of age who self-identified as Black in the United States and Canada. Multiple logistic regression assessed the relationship between health-seeking behaviors and ethnic origin, controlling for selected social and health-related factors. The mean age of the participants was 38.6 years, 31% were male, and 20% were unemployed. Sixty-one percent reported a very good or excellent health status, and 59.7% were not receiving treatment for chronic conditions. Advancing age (OR = 1.05, CI: 1.01–1.09), female gender (OR = 3.09, CI: 1.47–6.47), and unemployment (OR = 3.46, CI: 1.35–8.90) were associated with favorable health-seeking behaviors. Compared with the participants with graduate degrees, individuals with high school diplomas or less (OR = 3.80, CI: 1.07–13.4) and bachelor’s degrees (OR = 3.57, CI: 1.3–9.23) were more inclined to have engaged in favorable health-seeking behavior compared to those with graduate degrees. Across the Black communities in our sample, irrespective of ethnic origins or country of birth, determinants of health-seeking behavior were age, gender, employment status, and educational attainment.

## 1. Introduction

One of the key factors examined in the social determinants of health literature is health-seeking behavior [1]. Health seeking is the process of engaging in actions that an individual believes will resolve their health problem or illness [2]. The importance of health-seeking behavior for population health cannot be understated. Research is clear that health-seeking behavior and healthcare utilization are persistently poorer among under-represented populations [3]. Public health workers are constantly working to find new ways to increase health-seeking behavior and healthcare utilization to reduce health inequities in the United States [4]. In the United States, public health and policy makers are campaigning to increase health-seeking behavior and health service utilization to reverse the current downward trend in health-seeking behavior [5]. Healthcare utilization is one of the primary predictors of a healthy population and a center point of Healthy People 2030 [6]. Health-seeking behavior involves a social process that is influenced not just by one’s own personal views and standards but also by those of others via interactions with social networks [4]. Health-seeking behavior, which unsurprisingly has a direct impact on health status, includes the process of selecting a specific health treatment and broader patterns of decision-making behavior [7,8]. Other studies revealed that health-seeking behavior is influenced by a diverse set of gender, social, cultural, and economic factors as well as disease patterns [9]. African Americans experience higher rates of chronic illnesses such as hypertension, diabetes, and obesity than White Americans [3]. This calls to question whether the processes of seeking healthcare or not seeking healthcare can be influenced by socio-cultural and community factors [10]. Research found that health-seeking behavior varies across race/ethnicity and by source of information [11]. Further, it was found that racial differences in health-seeking behavior exist in the United States and that differences in health-seeking behavior by race are predicted by who is delivering health information for Black, White, and Hispanic individuals [12]. It is vital that we properly understand the impact of demographic and health factors on health-seeking behavior [13].

Racial and ethnic disparities in healthcare access and utilization in the United States have posed a persistent challenge. Numerous studies highlighted that racial and ethnic minorities tend to have fewer physician visits, lack regular primary care providers, and are less inclined to seek out health screenings [14,15,16]. Specifically, within ethnic minority groups, Black individuals are more reliant on emergency rooms as their primary source of healthcare and often delay seeking care to avoid out-of-pocket expenses such as deductibles. Moreover, compared to their counterparts, Black individuals are less likely to receive outpatient neurology visits or utilize behavioral health services, outpatient musculoskeletal care, or ophthalmology services [17,18,19,20,21].

Among the Black populations, African immigrants underutilize healthcare for various reasons. Common factors that hinder healthcare utilization in this subpopulation have been the cost of medical treatment, lack of insurance, having no access to free clinics, and language barriers [22,23,24]. Haitians reported the underutilization of healthcare services because of a cultural stigma surrounding mental health issues and mistrust of Western medicine [24]. Caribbean men report enabling factors such as adequate income, gainful employment, marital status, and health insurance in addition to factors such as mental health status as the most significant determinants of their willingness to utilize their usual sources of health care [25]. With specific reference to gender within Black communities, studies showed that women are less likely to be screened for depression and less likely to receive subsequent care, while men are less likely to receive mental health care [26]. Black men often delay seeking outpatient care or preventive health screenings because of medical mistrust, lack of insurance, and lack of access to the care [16]. In addition, Black women, compared to their White counterparts, are more likely to receive poor medical care, undergo unnecessary procedures, and experience discrimination when accessing medical care [27].

Healthcare utilization is linked to access to insurance, and the Affordable Care Act (ACA) has reduced disparities in healthcare utilization by making health insurance more affordable in the U.S. [28,29]. Studies, however, reported that after the ACA was implemented, certain racial/ethnic and gender-specific groups such as Black women and men have continued to experience increased rates of unmet needs and continue to comprise the most disadvantaged groups [30]. Another study that investigated the association between ACA enrollment, deductibles, healthcare utilization, and clinical outcomes for HIV patients found that Black and Hispanic individuals living with HIV experienced suboptimal HIV outcomes [31]. Thus, these mixed results highlight the need to examine healthcare utilization patterns among specific subgroups of racial and ethnic minorities. Past research showed that healthcare utilization is lower in Black communities; however, only a few studies explored the relationship between social determinants of health, health-seeking behavior, and healthcare utilization among specific subgroups in Black communities, including African, Afro-Caribbean, and other Black-identified groups [32,33]. Thus, our study contributes to filling this knowledge gap by examining how racial, ethnic, cultural, and social identities shape health-seeking behavior among African Americans, Afro-Caribbeans, and people of African descent in the United States and Canada. In particular, the study examines the determinants of health-seeking behavior among several key subgroups in the Black communities in the United States and Canada.

## 2. Methods

### 2.1. Data Collection/Study Design

We collected our data through the RHEALTH study, a multiyear cross-sectional study of Afro-Caribbean, African American, Afro-Canadian, and African respondents. A total of n = 293 Black-identifying respondents from the United States and Canada were recruited between 2017 and 2022, with a 2-year COVID pause. To be eligible for this study, the participants were required to be over the age of 18 years, be able to read, write, and understand English, and identify as Black. The decision to focus on ethnic subgroups of Black individuals rested on the fact that Black individuals are often treated as one category in research and yet have unique ethnic and subgroup differences that may influence their health behavior.

The RHEALTH study used multiple recruitment methods, including neighborhood canvassing, word of mouth, community events, and snowball sampling to identify a diverse sample of respondents. The respondents were told about the purpose of the study and its benefits for their community. The eligible participants were then provided with a QR code that linked them directly to an online self-administered survey with an access code. The survey contained 61 multiple-choice and open-ended questions related to Black identity, ethnic identity, perceived discrimination, and health conditions. An informed-consent form was provided online, describing the purpose of the study and compensation for participation. The participants who completed the online survey received an email with a link to access a USD 20 gift card. This study was approved by the institutional review board of the University of Washington and was granted exempt status due to its low or minimal risk to the respondents.

### 2.2. Outcome Variable

The outcome variable of interest was health-seeking behavior. Health-seeking behavior was classified based on the participants’ responses to the question “In the last 12 months, did you make an appointment for your health care at a doctor’s office or clinic as soon as you thought you needed it”? The responses options were “never”, “sometimes”, “usually”, and “always”. The outcome variable was further dichotomized into favorable health-seeking behavior—seeking care “usually/always” when needed—and non-favorable health-seeking behavior—seeking care “sometimes/never” when needed.

### 2.3. Exposure Variables

Ethnic origin was self-reported and classified as “Black American”, “Black African”, “Black Caribbean”, or “Black Canadian”.

The country of birth was assessed based on whether the participants were born in the U.S./Canada or not and was dichotomized as “yes” or “no”.

### 2.4. Other Variables

Gender was categorized as “female” or “male”. Age was included as a continuous variable. Education was classified as “high school/GED or less”, “some college”, “bachelor’s degree”, and “graduate degree”. The annual household income was categorized as <USD 40,000, USD 40,000–USD 59,999, USD 60,000–USD 89,999, and ≥USD 90,000. The marital status was defined as “married/living with a partner”, “never married”, or “separated/widowed/divorced”. Employment was categorized as “employed” or “unemployed”, regardless of the employment type. The health status was the self-reported perception of a respondent’s health and was categorized as “poor”, “fair”, “good”, “very good”, or “excellent”. The variable was later reclassified into “poor/fair”, “good”, and “very good/excellent”. Chronic disease included being treated for any of the following chronic conditions: heart disease, high blood pressure, diabetes, anemia or blood disease, arthritis, or chronic back pain. The variable was later dichotomized as “yes” if the participants were receiving treatment for any of these conditions at the time of the survey or “no” if they did not have any of these conditions. Insurance was reported as “no insurance”, “commercial”, “Medicaid”, “Medicare”, “Canadian Medicare”, “supplemental/private”, or “both Canadian Medicare and supplemental”. The variable was later recoded as “insured” or “uninsured”. If the participants were Canadian, the insurance response was indicated as “insured” because of the universal healthcare system in the country.

### 2.5. Statistical Analyses

The sociodemographic characteristics of the sample were described, stratifying the responses by health-seeking behavior as favorable or unfavorable. The continuous variables were reported using means (±SD), while the categorical variables were analyzed using the chi-squared (χ) test. Logistic regression was used to assess the odds of favorable health-seeking behavior within the sample population. The odds ratios were employed to measure the associations between exposure by group and the outcome of interest (health-seeking behavior). In the selection of references for the model, we utilized the normative category, such as employed versus unemployed, with employed serving as our normative reference. Alternatively, in cases where a normative group was not applicable, the largest category or the theoretically plausible referent group was selected as the reference. A Hosmer–Lemeshow test was conducted to assess the goodness of fit of the model. A Hosmer–Lemeshow statistic with a significance value greater than 0.05 suggests that the model adequately fits the data. Statistical significance was established as *p* < 0.05 for all statistical analyses. All analysis were performed using IBM SPSS Statistics for Windows, Version 28.0, released by IBM Corp in 2021, Armonk, NY, USA.

## 3. Results

In this analysis, we included 239 individuals from the United States and Canada. The average age of the participants was 38.6 years (±15.6), with 69.4% of them being women. Most participants identified as Black American, comprising 58.9% of the sample. The educational achievements varied, with 33.6% of the participants holding bachelor’s degrees, 26.2% having pursued some college education, and 21.0% having attained graduate degrees. The majority (80%) were employed, 44.3% of participants had never been married, and 31.9% reported a household income of less than USD 40,000. Regarding health, over half (60.7%) of the participants self-reported a good or excellent health status, and 59.7% claimed no history of chronic disease diagnosis. A significant portion, 72.5%, were native to either the United States or Canada. The majority (91.7%) of the respondents who answered the insurance question reported having some form of insurance or healthcare coverage.

Disparities in health-seeking behavior emerged across various demographic and health-related variables including age, gender, income, employment status, insurance coverage, and the presence of chronic diseases. Table 1 presents the association between sociodemographic factors and both favorable and non-favorable health-seeking behaviors. Older individuals (mean age 42.1 vs. 33.9, *p* < 0.001) exhibited a greater tendency toward a favorable health-seeking behavior. A higher proportion of women (80.8% vs. 54.6%; *p* < 0.001), unemployed individuals (28.1% vs. 9.7%; *p* < 0.001), and those with some form of insurance (98.7% vs. 84.6%; *p* = 0.001) exhibited a favorable health-seeking behavior. Conversely, individuals earning less than USD 40,000 annually (41.4% vs. 24.2%; *p* < 0.05) and those without any chronic condition (70.3% vs. 50%; *p* < 0.05) were more likely to report an unfavorable health-seeking behavior.

### Health-Seeking Behavior

Table 2 presents the results of a multivariate regression analysis of health-seeking behavior by various demographic and social determinants and perceived health status variables. Age was significantly associated with an increased propensity for seeking medical care. For each one-year increment in age, there was a corresponding 0.03-point increase in health-seeking behavior ([OR] = 1.03, 95% confidence interval [CI]: 1.01–1.06). This finding underscores the importance of age as a determinant in healthcare utilization patterns. Women exhibited a threefold greater likelihood of engaging in favorable health-seeking behavior compared to men (OR = 3.09, CI: 1.47–6.47). Similarly, unemployed individuals had greater inclination to seek care compared to employed individuals (OR = 3.46, CI: 1.35–8.90).

In our sample, we observed an inverse relation between educational level and health-seeking behavior. This implies that individuals with higher levels of education may be less inclined to engage in health seeking behavior. Compared with the participants who reported having earned graduate degrees, those who reported having earned bachelor’s degrees (OR = 3.57, 95% CI: 1.38–9.23) and individuals with a high school diploma or less (OR = 3.80, 95% CI: 1.07–13.4) were over three times more likely to exhibit positive health-seeking behavior. Our analysis did not reveal a statistically significant relationship between self-rated health and health-seeking behavior. Due to significant collinearity with self-rated health, the variable “being treated for chronic illnesses” was omitted from the full multivariable regression model. In a sensitivity analysis, however, being treated for chronic diseases showed no association with health-seeking behavior. Furthermore, after controlling for all other covariates, no statistically significant associations were found between ethnic origin or place of birth, household income, self-rated health, and health-seeking behavior.

## 4. Discussion

Health-seeking behavior is often used as a proxy to understand how individuals engage with healthcare systems. Recognizing the multifaceted nature of health-seeking behavior, which encompasses a spectrum of actions individuals undertake to safeguard or augment their well-being, including but not limited to seeking medical care, we sought to illuminate the factors shaping these behaviors. Our inquiry was driven by the understanding that such behaviors are profoundly influenced by the socio-cultural, economic, and demographic milieu in which individuals are situated [34]. In this study, we explored the intricacies of health-seeking behavior in Black populations in the United States and Canada, disaggregating the Black racial category based on ethnic origin and place of birth. Our study highlights three major findings. First, we found that within our sampled population, a person’s ethnic origin or country of birth did not emerge as a determinant factor in predicting whether that person had the propensity to engage in favorable health-seeking behavior. Second, our study revealed a notable disparity in health-seeking behavior between Black men and women across diverse ethnic backgrounds. Black men exhibited a higher prevalence of unfavorable health-seeking behavior compared to their female counterparts. Third, our study showed a compelling association between educational attainment and health-seeking behavior within the black population. Contrary to expectations, individuals with lower levels of education demonstrated a higher likelihood of engaging in a favorable health-seeking behavior compared to those with higher educational credentials.

While cultural factors undoubtedly shape perceptions of health and the importance attributed to seeking medical care, we did not find differences in health-seeking behavior based on ethnic origins or country of birth in our sample. Previous research highlighted the influence of socio-cultural factors, income disparities, and a pervasive distrust of the U.S. healthcare system among Black Americans, contributing to variations in health-seeking behaviors [35]. Similarly, a review conducted by Omenka et al. shed light on the underutilization of healthcare services within Black communities of African origin, citing barriers such as traditional beliefs, linguistic discordance, cultural competency challenges, the complexity of the U.S. healthcare system, and a lack of trust in the U.S. system [36]. Concerning the Caribbean population, existing studies offer limited clarity on whether the factors contributing to health disparities within this demographic mirror those observed in other immigrant populations. For instance, while some studies suggested similarities, such as in a study investigating ethnic descent and the risk of hypertension, participants with hypertension among the Afro-Caribbean group exhibited higher healthcare utilization compared to other racial groups [37]. While the broader landscape remains relatively understudied and inconclusive, the importance of comparing health characteristics between individuals born in a host country and those born abroad has been emphasized [38,39]. One potential explanation for the findings of our study could be linked to the inclusion criteria, as we solely enrolled English speakers. This group might possess an elevated awareness of the nuances related to racial issues, potentially leading to similar attitudes towards doctors or medical institutions. Incorporating study tools in languages other than English could have accommodated a more diverse range of participant responses. Additional research is warranted to unpack the complexities and variations within and across ethnic and immigrant communities.

Our study confirmed a significant gender disparity, indicating that Black women were three times more likely than Black men to have engaged in favorable health-seeking behavior. This finding aligns with those reported in numerous studies demonstrating a wide gender disparity within the United States [40,41]. According to the Centers for Disease Control and Prevention (CDC), men face a higher risk of death than women in every age group, experiencing 1.6 times higher mortality for all causes, 1.8 times higher mortality for heart diseases, 1.4 times higher mortality for cancers, and 2.4 times higher mortality for accidents [42]. These statistics and disparities are even worse for men of color, amplifying the health disparities within this demographic [43]. This gender discrepancy sheds light on the importance of tailored interventions and targeted outreach efforts to address the unique healthcare needs and barriers faced by Black men.

We found a significant association between age and health-seeking behavior, with advancing age correlating with a greater propensity of engaging in favorable health-seeking practices. This study aligns with research by the Institute of Medicine, which identified a relationship between older adults and a higher rate of chronic diseases compared to their younger counterparts, thus validating our observations [44]. However, our study did not find a significant relationship between chronic disease and health-seeking behavior. One plausible explanation for this discrepancy could be the relatively youthful composition of our sample population, with a mean age of 38 years. Thus, the manifestation and impact of chronic diseases may not have fully materialized within this cohort, potentially attenuating the observed relationship between chronic disease and health-seeking behavior.

Empirical evidence demonstrates that achieving higher education positively influences health through various pathways. These include direct effects on health, along with indirect effects mediated by better work and improved economic situations, bolstered social and psychological assets, and the adoption of healthier lifestyles [45]. Education also plays a great role in the utilization of healthcare services. However, in our study, we observed that compared to Black people with graduate degrees, individuals holding bachelor’s degrees or possessing a high school education or less were more inclined to seek care when needed. This trend diverges from that reported in the established literature, which typically demonstrates a positive association between educational level and health-seeking behavior [46]. It may also vary depending on the type of care sought by individuals. For instance, a study by Van der Vlegel et al. (2021) examined healthcare usage by type and found a nuanced pattern: individuals with a lower educational attainment were more likely to seek outpatient rehabilitation and psychological care and less likely to seek physical therapy than their more educated counterparts [47]. It is plausible that the observed inverse relationship between education and healthcare utilization in our study could be attributed to the healthier habits typically associated with higher education levels. Educated individuals are often more proactive in adopting healthier lifestyles and managing their health, which potentially leads to fewer visits to healthcare providers. This emphasizes the importance of understanding the complex interaction between education, health behaviors, and the use of healthcare services. We also observed an inverse relationship between employment status and health-seeking behavior, with unemployed individuals being almost three and a half times more likely to seek medical care when needed. It would be valuable to investigate whether unemployment provides individuals with greater flexibility and time to schedule appointments with doctors, particularly if they are covered by insurance.

The relationship between insurance and health-seeking behavior is complex. While insurance provides financial coverage for medical expenses, various factors may influence the timing and how individuals seek and access healthcare services. In our univariate analysis, a statistically significant relationship between insurance and health-seeking behavior was observed. However, it is important to acknowledge that our insurance variable exhibits substantial missing data. The majority of the respondents who answered the question reported having insurance, indicating a possible systematic difference between the missing data and the observed values. Additionally, the participants from Canada benefited from universal health insurance coverage. This might have introduced bias into our insurance data, potentially resulting in an overrepresentation of individuals with insurance in our sample. Consequently, we excluded insurance from the multiple regression analysis. However, our findings indicate that being from Canada or the U.S. did not exhibit a significant association with health-seeking behavior. While having healthcare coverage undoubtedly plays a pivotal role in facilitating access to services, particularly in the United States where it significantly impacts individuals’ ability to afford medical care, its influence on health-seeking behavior within our study was not fully explored. However, it is worth noting that other factors beyond insurance status, such as cultural beliefs, socioeconomic status, or perceived healthcare quality, exert greater influence on individuals’ decisions regarding when and how to seek medical care. Further studies are needed to gather more comprehensive and representative insurance data and enhance our understanding of the interplay between insurance coverage and health-seeking behavior.

Against the backdrop of persistent racial challenges, there has been a resurgence in efforts to dissect and address the multifaceted challenges faced by Black individuals in accessing healthcare. The COVID-19 pandemic, amplifying pre-existing disparities, has starkly highlighted vulnerabilities entrenched within marginalized communities and thus the need for a critical examination of their trust in the healthcare system. In this context of heightened racial awareness, future research could offer invaluable insights by probing the nuanced dynamics of medical mistrust and the enduring legacy of historical trauma within healthcare interactions, with a particular focus on Black populations. Moreover, it is imperative for future studies to comprehensively explore additional social determinants of health including geographic location, physical environment, cultural and social norms, transportation accessibility, and social support networks.

The strength of this study lies in its collection of data from diverse ethnic subgroups that constitute Black communities across the United States and Canada. This inclusive sampling allowed for a thorough examination of the intra-group differences in health-seeking behavior. However, while the outcome variable, i.e., scheduling a doctor’s appointment when deemed necessary, is certainly pertinent, its scope may be limited, as it focuses primarily on a singular action within the broad spectrum of healthcare engagement. A more comprehensive approach might encompass various dimensions of proactive health management, including preventive measures, timely regular screenings, and adherence to treatment plans. We also used convenience sampling, which poses a constraint in the generalizability of our results [48]. Additionally, this study relied on self-reported data, which are susceptible to the social desirability bias. However, such data have demonstrated utility in general health measures, particularly in understanding people health status [48]. Despite these limitations, our study provides valuable insights into the health-seeking behaviors within Black communities, shedding light on important dynamics that warrant further exploration.

## 5. Conclusions

This cross-sectional study examined differences in health-seeking behavior among diverse ethnic subgroups within Black populations across the United States and Canada. The findings revealed that, irrespective of ethnic origins or country of birth, age, gender, employment status, and educational attainment significantly influenced the health-seeking behavior within these communities. Recognizing the multifaceted nature of health-seeking behavior, it is crucial to explore the complex interplay of socioeconomic factors, cultural influences, and individual attitudes towards healthcare to gain a deeper understanding of how these elements shape healthcare utilization patterns within Black communities. Understanding the intersection of these factors and their impact on healthcare utilization can offer valuable insights for developing targeted interventions to enhance access to healthcare and mitigate health disparities. Future studies should adopt a more nuanced approach to delve into the dynamics of medical mistrust before and after pivotal events such as the COVID-19 pandemic and the racial reckoning.

## Figures and Tables

**Table 1:** Baseline characteristics of the survey respondents by health-seeking behavior.

		Unfavorable n (%)	Favorable n (%)	Chi-Squared *p*-Value
Age, Years (n = 239)	Mean (±SD)	33.86 (±10.9)	42.07 (±17.8)	**<0.001**
Gender (n = 222)	Female	53 (54.6)	101 (80.8)	**<0.001**
	Male	44 (45.1)	24 (19.2)	
Ethnic Group (n = 234)	Black/African American	59 (57.3)	79 (60.3)	0.104
	Black Caribbean	12 (11.7)	24 (18.3)	
	Black African	27 (26.2)	19 (14.5)	
	Black Canadian	5 (4.8)	9 (6.9)	
Country (n = 227)	USA	73 (82.0)	116 (84.1)	0.688
	Canada	16 (18.0)	22 (15.9)	
Born in U.S. or Canada (n = 233)	No	27 (26.5)	37 (28.2)	0.763
	Yes	75 (73.5)	94 (71.8)	
Educational Status (n = 229)	High School/GED or Less	25 (24.3)	19 (15.1)	0.180
	Some College	28 (27.2)	32 (25.4)	
	Bachelor’s Degree	28 (27.2)	49 (38.9)	
	Graduate Degree	22 (21.3)	26 (20.6)	
Household Income (n = 226)	<USD 40,000	42 (41.1)	30 (24.2)	**0.018**
	USD 40,000–USD 59,999	23 (22.6)	27 (21.7)	
	USD 60,000–USD 89,999	23 (22.6)	34 (27.4)	
	USD 90,000 or More	14 (13.7)	33 (26.6)	
Marital Status (n = 226)	Never Married	50 (49.0)	50 (40.3)	0.321
	Separated/Divorced/Widowed	11 (10.8)	20 (16.1)	
	Married	41 (40.2)	54 (43.6)	
Employment Status (n = 231)	Unemployed	10 (9.7)	36 (28.1)	**<0.001**
	Employed	93 (90.3)	92 (71.9)	
Insurance (n = 156)	No	12 (15.4)	1 (1.3)	**0.001**
	Yes	66 (84.6)	77 (98.7)	
Self-Rated Health (n = 224)	Very Good/Excellent	67 (67.7)	69 (55.2)	0.163
	Poor/Fair	12 (12.1)	20 (16.0)	
	Good	20 (20.0)	36 (28.8)	
Chronic Disease * (n = 154)	No	52 (70.3)	40 (50.0)	**0.010**
	Yes	22 (29.7)	40 (50.0)	

Data are shown as n (%) except when indicated otherwise. SD: standard deviation. Bold: chi squared. *p* < 0.05. * Chronic disease includes being treated for any of the following chronic conditions: heart disease, high blood pressure, diabetes, anemia or blood disease, arthritis, or chronic back pain.

**Table 2:** Logistic regression analyses of the association of health-seeking behavior with sociodemographic and health-related factors (n = 199).

	Favorable Health-Seeking Behavior (n = 199)	
Characteristics	OR (95%Cl)	*p*-Value
Survey Country		
U.S.	(Ref)	
Canada	0.79 (0.15–4.10)	0.792
Ethnic Group		
Black American	(Ref)	
Black Caribbean	1.05 (0.29–0.73)	0.944
Black African	0.71 (0.24–2.11)	0.536
Black Canadian	1.73 (0.21–14.23)	0.608
Age	1.03 (1.01–1.06)	**0.029**
Gender		
Male	(Ref)	
Female	3.09 (1.47–6.47)	**0.003**
Employment Status		
Employed	(Ref)	
Unemployed	3.46 (1.35–8.90)	**0.010**
Educational Attainment		
Graduate Degree	(Ref)	
Bachelor’s Degree	3.57 (1.3–9.23)	**0.009**
Some College	2.48 (0.89–6.93)	0.084
High School/GED or Less	3.80 (1.07–13.4)	**0.038**
Household Income		
<USD 40,000	(Ref)	
USD 40,000–USD 59,999	1.85 (0.74–4.61)	0.186
USD 60,000–USD 89,999	1.80 (0.74–4.36)	0.192
≥USD 90,000	2.78 (0.75–8.11)	0.062
Self-Rated Health		
Very Good/Excellent	(Ref)	
Good	1.75 (0.79–3.87)	0.167
Poor/Fair	1.71 (0.68–4.30)	0.258
Born in U.S. or Canada		
No	(Ref)	
Yes	1.43 (0.47–4.37)	0.527

Bold: *p* < 0.05; OR, odds ratio; CI, confidence interval.

## Data Availability

The data are available upon reasonable request.
